# Effects of Parkinson’s disease on survival in cancer survivors: a retrospective, multicentre cohort study in Japan

**DOI:** 10.1093/braincomms/fcaf347

**Published:** 2025-09-13

**Authors:** Kanako Asai, Yasufumi Gon, Yasuyoshi Kimura, Toshitaka Morishima, Hideki Mochizuki, Isao Miyashiro

**Affiliations:** Department of Neurology, Osaka University Graduate School of Medicine, Suita, Osaka 565-0871, Japan; Cancer Control Center, Osaka International Cancer Institute, Osaka-shi, Osaka 541-8567, Japan; Department of Neurology, Osaka University Graduate School of Medicine, Suita, Osaka 565-0871, Japan; Cancer Control Center, Osaka International Cancer Institute, Osaka-shi, Osaka 541-8567, Japan; Department of Medical Innovation, Academic Clinical Research Center, Osaka University Hospital, Suita, Osaka 565-0871, Japan; Department of Neurology, Osaka University Graduate School of Medicine, Suita, Osaka 565-0871, Japan; Cancer Control Center, Osaka International Cancer Institute, Osaka-shi, Osaka 541-8567, Japan; Department of Neurology, Osaka University Graduate School of Medicine, Suita, Osaka 565-0871, Japan; Cancer Control Center, Osaka International Cancer Institute, Osaka-shi, Osaka 541-8567, Japan

**Keywords:** Parkinson disease, cancer survivors, mortality, cohort study

## Abstract

Aging populations and advances in medical care have contributed to an increase in the number of patients with Parkinson’s disease and cancer survivors. However, the effects of Parkinson’s disease on the survival of patients with cancer remain unclear. We aimed to investigate the effect of Parkinson’s disease on the prognosis of patients with cancer. This retrospective multicentre cohort study used data from the Osaka Cancer Registry, which was linked to administrative data obtained between 2010 and 2015 in Japan. Patients with cancer who were followed up for 5 years after cancer diagnosis were categorized into Parkinson’s disease and non- Parkinson’s disease groups according to the presence of comorbid Parkinson’s disease. We compared survival between the two groups using Kaplan–Meier analyses. Cox proportional-hazard models were used to assess the hazard ratio and 95% confidence interval of Parkinson’s disease for all-cause mortality. Propensity-score matching was used to validate the analysis and address differences in clinical characteristics between the groups. The cohort included 118 999 patients with cancer and 1049 patients (0.9%) with comorbid Parkinson’s disease. The patients in the Parkinson’s disease group were older and had lower mobility than those in the non- Parkinson’s disease group. Kaplan–Meier analysis revealed that the 5-year survival rate after cancer diagnosis was significantly worse in the Parkinson’s disease group than in the non- Parkinson’s disease group (54% versus 70%, *P* < 0.001). Comorbid Parkinson’s disease was independently associated with 5-year mortality [crude hazard ratio (95% confidence interval), 1.72 (1.56–1.88), *P* < 0.001]. This association remained significant after adjusting for potential confounders [adjusted hazard ratio (95% confidence interval), 1.37 (1.25–1.51), *P* < 0.001]. When the survival analysis was stratified by mobility, no intergroup difference in prognosis was observed among patients with impaired mobility. However, among patients with independent mobility, patients in the Parkinson’s disease group had a worse prognosis than those in the non- Parkinson’s disease group [crude hazard ratio (95% confidence interval), 1.74 (1.42–2.13), *P* < 0.001]. Similar results were observed after propensity-score matching. Patients with cancer and comorbid Parkinson’s disease had a worse prognosis than those without comorbid Parkinson’s disease. The effect varied depending on mobility. These findings highlight the importance of carefully managing Parkinson’s disease symptoms and maintaining mobility in cancer patients with Parkinson’s disease, as this could potentially improve their prognosis and survival outcomes.

## Teaser

In this multicentre cohort study, Asai *et al*. reported that cancer patients with Parkinson’s disease had a significantly worse 5-year survival rate than those without Parkinson’s disease. The effect varied depending on mobility. These findings highlight the importance of managing Parkinson’s disease symptoms and maintaining mobility in cancer patients with Parkinson’s disease.

## Graphical Abstract

**Graphical Abstract:**
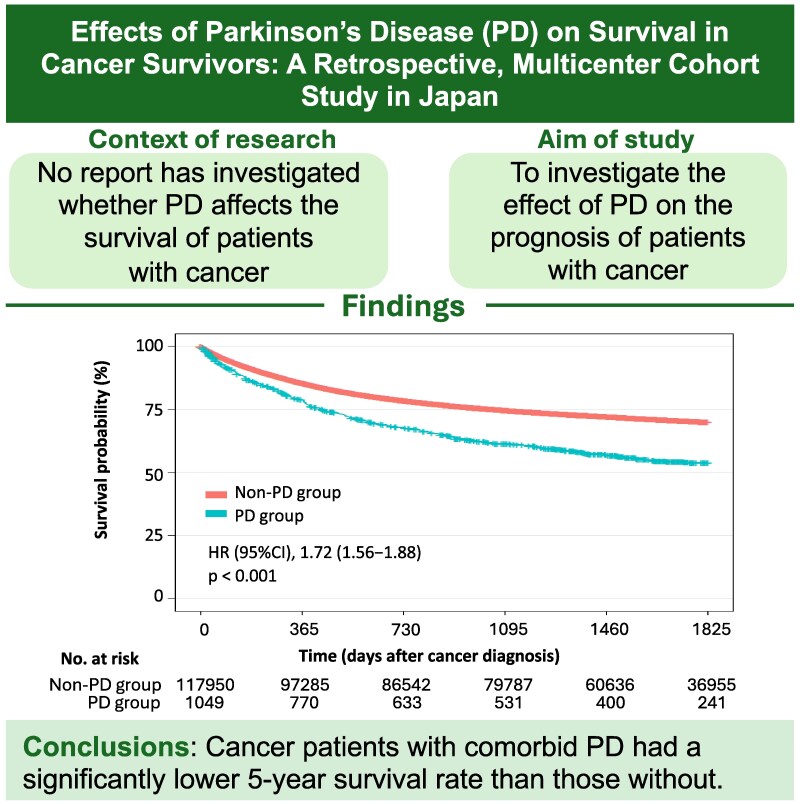

## Introduction

Parkinson’s disease is a progressive neurodegenerative disorder characterized by cardinal motor symptoms such as bradykinesia, rigidity and tremor, along with various non-motor symptoms, including cognitive impairment.^1^ With the aging of the global population, the number of patients with Parkinson’s disease has increased worldwide. The estimated number of individuals with Parkinson’s disease was ∼6 million in 2015 and is projected to exceed 12 million by 2040.^2-4^ Advances in treatment have improved the quality of life and prognosis for patients with Parkinson’s disease.^5^ However, the extended duration of illness heightens the risk of complications with other diseases.

Concurrently, the aging population and advancements in medical care have also contributed to an increase in cancer survivors.^6^ While previous studies have suggested that patients with Parkinson’s disease are less likely to develop cancer, the anticipated growth in the number of patients with Parkinson’s disease as well as cancer survivors may result in a substantial increase in individuals affected by both conditions.^7-9^ Regarding the relationship between Parkinson’s disease and cancer, previous studies have suggested an inverse association for the risk of developing the disease. Although no definitive mechanisms have been established to explain this inverse correlation, it may be attributed to fundamentally opposing biological mechanisms: enhanced cell death in Parkinson’s disease versus uncontrolled cell proliferation in cancer.^10^ In contrast, there are few reports on how the coexistence of Parkinson’s disease and cancer affects the clinical course of both disease once both have developed, specially whether the progression of Parkinson’s disease or cancer is altered by their comorbidity. A previous study examined the influence of Parkinson’s disease on the postoperative outcomes of patients with gastric and colorectal cancer, suggesting that Parkinson’s disease is associated with a low discharge rate to home.^11^ We reported in a previous study, using the Neoplasms and other causes of Death database, that cancer survivors are at high risk for Parkinson’s disease–related death as a cause of their death.^12^ However, no report has investigated whether Parkinson’s disease affects the prognosis of cancer survivors.

Japan has one of the world’s most aged populations. Since the population of older adults in Japan may represent a future scenario for other countries, investigating the clinical outcomes of Japanese individuals with Parkinson’s disease and cancer in clinical settings has become increasingly relevant. This study aimed to assess the prognostic impact of Parkinson’s disease comorbidity in cancer survivors, using a different dataset from the one we previously reported.^12^

## Materials and methods

### Study design and data source

This retrospective multicentre cohort study used data from the Osaka Cancer Registry (OCR) in combination with the Diagnosis Procedure Combination (DPC) data in Japan. The study cohort consisted of patients who were diagnosed with cancer and registered in the OCR between January 2010 and December 2015. The OCR has been operating since 1962 and covers over 8 million residents in the Osaka Prefecture.^13^ Cancer-related information was obtained from the OCR.^13^ Comorbidity and medication data were collected from the designated cancer care hospitals’ DPC data. The diagnoses in the DPC are registered using International Classification of Disease 10th (ICD-10) Edition codes. By merging the OCR and DPC data, we constructed the OCR-DPC database for research purposes.^14-16^ The OCR-DPC database was created in collaboration with the Council for Coordination of Designated Cancer Care Hospitals in Osaka. The data used in this study were collected from 36 hospitals and included >50% of the patients with cancer in Osaka Prefecture. All codes for disease diagnoses and medications used in this study are provided in Supplementary Tables 1 and 2. The study participants were newly diagnosed cancer patients registered with the OCR. Patients aged <18 years at cancer diagnosis and those with missing DPC data were excluded from the study. After applying the exclusion criteria, a total of 118 999 patients with cancer were included in the final cohort. Of these, 1049 (0.9%) patients were assigned to the Parkinson’s disease group, whereas 117 950 (99.1%) were assigned to the non- Parkinson’s disease group.

### Clinical variables

The following baseline variables were obtained from the OCR-DPC database: sex, age at cancer diagnosis, body mass index, smoking history, Charlson Comorbidity Index (CCI), Barthel Index, cancer type, years of cancer diagnosis, stage at cancer diagnosis and cancer treatment (defined as receipt of cancer surgery, chemotherapy or radiotherapy; each categorized as yes or no). Age at cancer diagnosis was grouped into three categories: < 65, 66–74 and ≥75 years. Body mass index was divided into three groups according to World Health Organization criteria: < 18.5, 18.5–25 and >25.^17^ The CCI was grouped into three categories: 0–2, 3–4 and ≥5.^18^ Since the severity indices of Parkinson’s disease [e.g. Hoehn and Yahr scale (HY)] were not included in the OCR-DPC database, we estimated the patients’ motor symptoms (mobility) using the mobility score of the Barthel Index^19^ and classified them into the following three groups: independent (mobility score of 15, corresponding to HY stages 1–3), moderate immobility (mobility score of 10, corresponding to HY stage 4) and severe immobility (mobility score of 0 or 5, corresponding to HY stage 5). Since most anti–Parkinson’s disease treatments are oral medications, we hypothesized that cancers affecting the oral administration route would have a negative influence on the management of Parkinson’s disease symptoms, resulting in an increased risk of mortality.^12^ To account for the differences in the effects of such cancers from those of cancers that do not affect the oral administration route, we categorized the cancers into the following two groups: mouth-to-stomach cancers (including lip, oral cavity, pharynx, oesophagus and stomach cancers) and others. The stage at cancer diagnosis was classified into six categories: (i) intraepithelial (abnormal cells are present but have not spread to nearby tissues), (ii) localized (cancer is limited to the organ where it originated, with no sign of spread), (iii) regional (lymph node metastasis [cancer has spread to regional lymph nodes] and/or infiltration to adjacent organs [cancer has spread to nearby tissues or organs]), (iv) distant metastasis (cancer has metastasized to distant parts of the body), (v) not applicable (the extents of leukaemia and multiple myeloma are registered as ‘not applicable’ in the Japanese cancer registry) and (vi) unstaged (insufficient information to determine the stage). In the OCR, cancer treatment refers to the first treatment administered to a patient diagnosed with cancer that continues until the completion of the treatment plan. For haematologic malignancies, initial therapy refers to all therapies administered until the induction of initial remission.

### Ascertainment of Parkinson’s disease cases

Parkinson’s disease was defined by an ICD-10 code for Parkinson’s disease (G20) or a prescription of anti-parkinsonian medications (levodopa, dopamine agonists and monoamine oxidase inhibitor B).^11,20^ Patients with other neurodegenerative disorders (ICD-10 codes: G01-19, G21-99) were excluded. To account for survival bias, only patients who met the criteria for Parkinson’s disease within 6 months before or after their cancer diagnosis date were assigned as having Parkinson’s disease.

### Follow-up duration and outcomes

The observation period was 5 years after the cancer diagnosis. Information on deaths during the follow-up period was also collected. Patients who had a second cancer diagnosis or were lost to follow-up were censored. Death was defined as death due to any cause.

### Statistical analysis

All data are presented as median and interquartile range or counts and percentages. First, we classified the patients into two groups according to the presence of PD and compared the baseline characteristics between the Parkinson’s disease and non- Parkinson’s disease groups. Values were compared using the Mann–Whitney U-test for continuous variables and the chi-square test for categorical variables. Survival curves were generated using the Kaplan–Meier method, and survival differences between groups were assessed using the log-rank test. Because the prognosis of patients with Parkinson’s disease can be influenced by their motor symptoms, we also plotted survival curves stratified by patient mobility. Finally, hazard ratios (HRs) and 95% confidence intervals (CIs) for mortality were calculated using univariable and multivariable Cox proportional-hazard models. The following variables were considered potential confounders: sex, age at cancer diagnosis, body mass index, smoking history, mobility status, CCI, cancer stage, cancer type, cancer treatment and year of cancer diagnosis. The missing values were imputed using multiple imputation techniques.

To address the differences in clinical characteristics between the Parkinson’s disease and non- Parkinson’s disease groups, we performed propensity-score matching analysis. This method aimed to identify a subgroup within the original cohort that exhibited baseline characteristics similar to those of the control group. The propensity score was computed using a multivariable logistic regression model, with the dependent variable being the presence or absence of Parkinson’s disease as a function of baseline variables, including sex, age at cancer diagnosis, smoking history, CCI, mobility status, cancer stage, cancer type, cancer treatment and years since cancer diagnosis. One-to-one nearest neighbour matching was conducted using a calliper, which was set to 0.25 times the standard deviation of the logit of the predicted probability of Parkinson’s disease computed using the multivariable logistic regression model. The standardized difference was calculated to evaluate the covariate balance, with a value < 0.10 regarded as balanced.

Statistical analyses were performed using R software (https://cran.r-project.org/). The level of significance was set at *P* < 0.05.

### Standard protocol approvals, registrations and patient consents

This study was approved by the Institutional Review Board of Osaka University Hospital (approval number: 22845). The need for written informed consent was waived owing to the retrospective study design. OCR data were obtained in accordance with the Act on the Promotion of Cancer Registries.

## Results

Table 1 shows the baseline characteristics of the groups (see Table 1 in Supplementary Data 1 for analysis code). In comparison with patients in the non- Parkinson’s disease group, those in the Parkinson’s disease group were older, had a lower body mass index and exhibited a lower prevalence of smoking. The CCI score was higher in the Parkinson’s disease group than in the non–Parkinson’s disease group. The mobility scores were lower in the Parkinson’s disease group than in the non–Parkinson’s disease group. The Parkinson’s disease group also showed a lower proportion of patients receiving cancer treatment than the non–Parkinson’s disease group.

**Table 1:** Baseline characteristics of patients with cancer with and without Parkinson’s disease

Variables	Parkinson’s disease group (*n* = 1049)	Non–Parkinson’s disease group (*n* = 117 950)	*P* value
Female, *n* (%)	430 (41)	50 281 (43)	0.29
Age, years (interquartile range)	73 (65–79)	70 (62–77)	<0.001
<65	239 (23)	38 420 (33)	
≥65 and <75	345 (33)	41 465 (35)	
≥75	465 (44)	38 065 (32)	
Body mass index, *n* (%)			0.01
<18.5	165 (16)	15 349 (13)	
≥18.5 and <25	614 (59)	75 394 (64)	
≥25	224 (21)	24 036 (20)	
Missing	46 (4)	3171 (3)	
Smoking, *n* (%)	332 (32)	45 428 (39)	<0.001
Charlson Comorbidity Index, *n* (%)			<0.001
0–2	422 (40)	71 037 (60)	
3–4	359 (34)	22 183 (19)	
≥5	266 (25)	24 431 (21)	
Missing	2 (0)	299 (0)	
Mobility, *n* (%)			<0.001
Independent	658 (63)	99 932 (85)	
Moderate	116 (11)	5496 (5)	
Severe	255 (24)	11 153 (9)	
Missing	20 (2)	1369 (1)	
Cancer stage, *n* (%)			<0.001
Intraepithelial	59 (6)	11 855 (10)	
Localized	452 (43)	50 250 (43)	
Regional	271 (26)	27 842 (24)	
Distant	182 (17)	21 159 (18)	
Not applicable	31 (3)	2882 (2)	
Unstaged	54 (5)	3962 (3)	
Cancer type, *n* (%)			0.026
Mouth-to-stomach	215 (20)	20 949 (18)	
Others	834 (80)	97 001 (82)	
Cancer treatment, *n* (%)			
Cancer surgery	640 (61)	76 760 (65)	0.006
Chemotherapy	289 (28)	40 857 (35)	<0.001
Radiotherapy	107 (10)	14 454 (12)	0.043
Year of cancer diagnosis, *n* (%)			0.002
2010	41 (4)	4121 (3)	
2011	85 (8)	8568 (7)	
2012	167 (16)	14 645 (12)	
2013	213 (20)	25 193 (21)	
2014	286 (27)	31 697 (27)	
2015	257 (24)	33 726 (29)	

**Figure 1:**
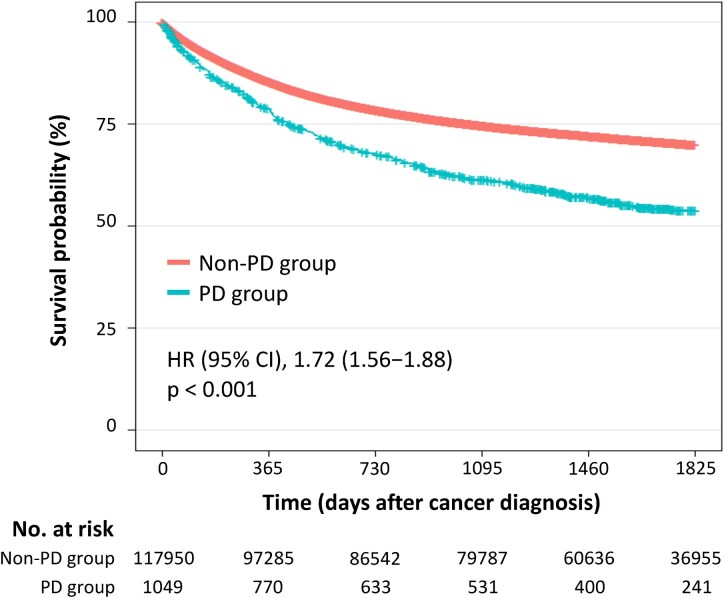
**Kaplan–Meier survival curves stratified by the presence of Parkinson’s disease.** Kaplan–Meier survival curves for the Parkinson’s disease and non–Parkinson’s disease groups. The number of patients was 1049 in the Parkinson’s disease group and 117 950 in the non–Parkinson’s disease group.

Figure 1 (see Fig. 1 in Supplementary Data 1 for analysis code) shows the 5-year survival rate after cancer diagnosis. Patients in the Parkinson’s disease group had significantly worse survival rates than those in the non–Parkinson’s disease group (54% versus 70%, log-rank *P* < 0.001). The results of the univariable and multivariable Cox regression analyses are presented in **Table 2** (see Table 2 in Supplementary Data 1 for analysis code). Parkinson’s disease was significantly associated with 5-year mortality among cancer patients [crude HR (95% CI), 1.72 (1.56–1.88), *P* < 0.001]. This association remained significant even after adjusting for potential confounders [adjusted HR (95% CI), 1.37 (1.25–1.51), *P* < 0.001]. Patients with mouth-to-stomach cancers, which may affect oral medication for Parkinson’s disease treatment, had an ∼1.2-fold higher risk of mortality than those with other types of cancer. In terms of Parkinson’s disease severity, patients with impaired mobility had a higher risk of mortality than those who could move independently. Patients who were recently diagnosed with cancer during the cohort period had a lower mortality risk.

**Table 2:** Hazard ratios for mortality determined using cox proportional-hazard models

Variables	Univariable		Multivariable^a^	
	HR (95% CI)	*P* value	HR (95% CI)	*P* value
Parkinson’s disease	1.72 (1.56–1.88)	<0.001	1.37 (1.25–1.51)	<0.001
Male	1.49 (1.46–1.53)	<0.001	1.23 (1.20–1.26)	<0.001
Age, years				
<65	Reference		Reference	
≥65 and <75	1.47 (1.43–1.52)	<0.001	1.29 (1.26–1.33)	<0.001
≥75	2.38 (2.31–2.44)	<0.001	1.97 (1.91–2.03)	<0.001
Body mass index				
<18.5	1.86 (1.77–1.87)	<0.001	1.41 (1.37–1.45)	<0.001
≥18.5 and <25	Reference		Reference	
≥25	0.77 (0.75–0.80)	<0.001	0.86 (0.84–0.89)	<0.001
Smoking	1.15 (1.13–1.18)	<0.001	1.02 (1.00–1.05)	0.06
Charlson Comorbidity Index				
<3	Reference		Reference	
≥3 and <5	2.45 (2.38–2.52)	<0.001	1.60 (1.55–1.65)	<0.001
≥5	7.60 (7.41–7.79)	<0.001	2.40 (2.30–2.50)	<0.001
Mobility				
Independent	Reference		Reference	
Moderate	3.01 (2.90- 3.13)	<0.001	1.38 (1.32–1.44)	<0.001
Severe	3.27 (3.18–3.36)	<0.001	1.42 (1.37–1.47)	<0.001
Cancer stage				
Intraepithelial	Reference	<0.001	Reference	
Localized	2.25 (2.10–2.43)	<0.001	1.58 (1.46–1.71)	<0.001
Regional	6.62 (6.15–7.13)	<0.001	4.49 (4.15–4.90)	<0.001
Distant	22.80 (21.18–24.53)	<0.001	6.19 (5.68–6.74)	<0.001
Not applicable	13.20 (12.10–14.41)	<0.001	4.95 (4.50–5.44)	<0.001
Unstaged	15.53 (14.29–16.88)	<0.001	6.22 (5.69–6.81)	<0.001
Cancer type				
Mouth-to-stomach	1.12 (1.09–1.15)	<0.001	1.23 (1.20–1.27)	<0.001
Cancer treatment				
Cancer surgery	0.24 (0.23–0.24)	<0.001	0.51 (0.50–0.53)	<0.001
Chemotherapy	2.19 (2.15–2.24)	<0.001	1.08 (1.05–1.11)	<0.001
Radiotherapy	1.09 (1.05–1.12)	<0.001	0.87 (0.84–0.90)	<0.001
Year of cancer diagnosis				
2010	Reference		Reference	
2011	0.92 (0.87–0.97)	<0.001	0.98 (0.93–1.04)	0.53
2012	0.84 (0.80–0.88)	<0.001	0.93 (0.88–0.97)	0.003
2013	0.50 (0.47–0.52)	<0.001	0.57 (0.54–0.60)	<0.001
2014	0.45 (0.43–0.48)	<0.001	0.53 (0.51–0.56)	<0.001
2015	0.36 (0.34–0.37)	<0.001	0.44 (0.42–0.46)	<0.001

^a^Adjusted for age, sex, body mass index, smoking history, Charlson comorbidity index, mobility, cancer stage, cancer treatment and year of cancer diagnosis.

Missing values were for Charlson Comorbidity Index and mobility.

CI, confidence interval; HR, hazard ratio.

**Figure 2:**
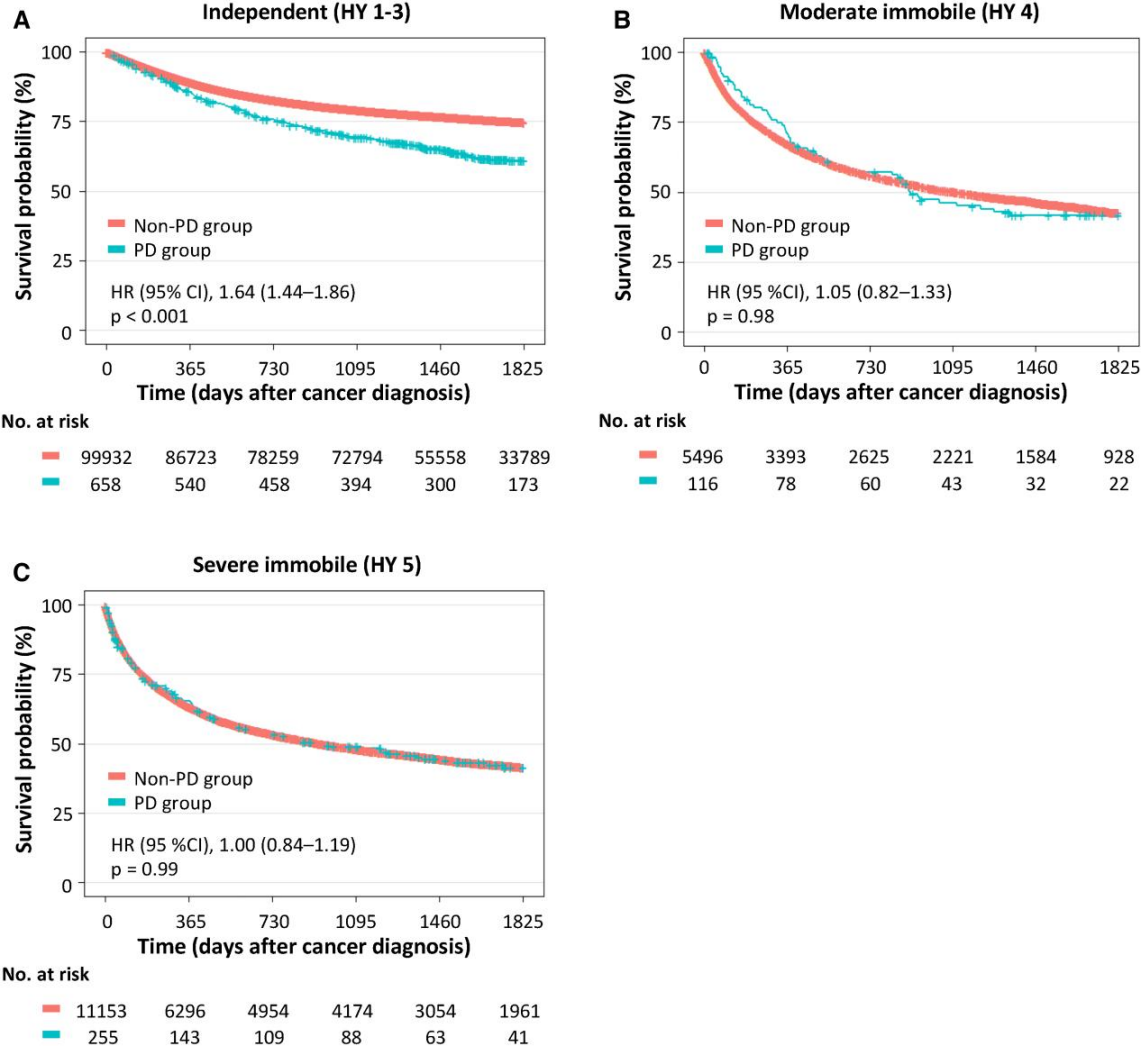
**Kaplan–Meier survival curves stratified by mobility status.** Kaplan–Meier survival curves for the Parkinson’s disease and non–Parkinson’s disease groups based on mobility status: independent mobility (**A**), moderate immobility (**B**) and severe immobility (**C**). The number of patients was 658 in the Parkinson’s disease group and 99 932 in the non–Parkinson’s disease group in group A and 116 in the Parkinson’s disease group and 5496 in the non–Parkinson’s disease group in group B and 255 in the Parkinson’s disease group and 11 153 in the non–Parkinson’s disease group in group C. Among the patients with independent mobility, those in the Parkinson’s disease group showed poorer survival than those in the non–Parkinson’s disease group. For patients with moderate or severe immobility, survival rates were similar in the Parkinson’s disease and non–Parkinson’s disease groups.

As shown in Table 2, mobility was an independent prognostic factor for mortality. To analyse the relationship between Parkinson’s disease and mobility in detail, we stratified the Kaplan–Meier curves by mobility status [Fig. 2 (see Fig. 2 in Supplementary Data 1 for analysis code)]. Patients with Parkinson’s disease who showed independent mobility (corresponding to HY stages 1–3) had poorer survival than the patients with independent mobility in the non- Parkinson’s disease group [crude HR (95% CI), 1.74 (1.42–2.13), *P* < 0.001]. In contrast, the survival rate of patients with Parkinson’s disease and moderately to severely impaired mobility (corresponding to HY stages 4–5) was comparable to that of non- Parkinson’s disease patients with moderately to severely impaired mobility.

**Table 3** (see Table 3 in Supplementary Data 1 for analysis code) shows the patient background characteristics after propensity-score matching. All clinical variables were balanced between the two groups, with standardized mean differences of <0.10. The survival rates after cancer diagnosis [Fig. 3 (see Fig. 3 in Supplementary Data 1 for analysis code)] and the results stratified by mobility [Fig. 4 (see Fig. 4 in Supplementary Data 1 for analysis code)] remained consistent with the results of the analysis using the entire cohort.

**Figure 3:**
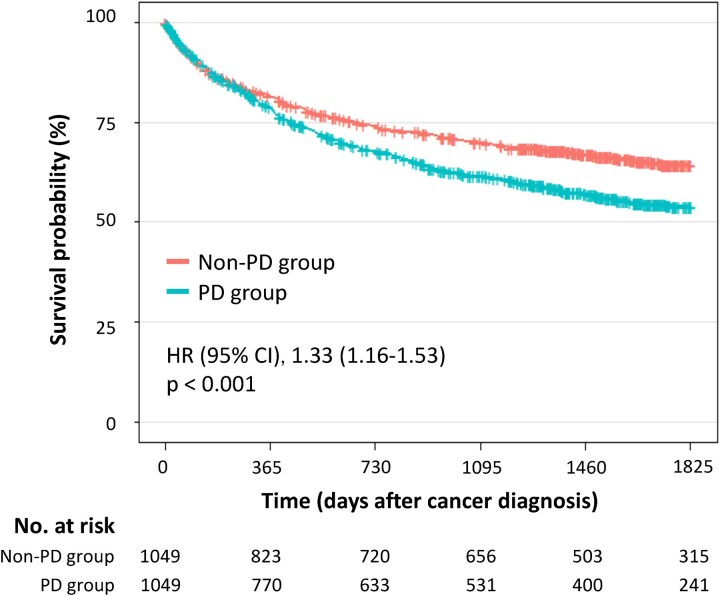
**Kaplan–Meier survival curves stratified by the presence of Parkinson’s disease after propensity score matching.** Kaplan–Meier survival curves for the Parkinson’s disease and non–Parkinson’s disease groups after propensity-score matching. The number of patients was 1049 in the Parkinson’s disease group and 1049 in the non–Parkinson’s disease group.

**Figure 4:**
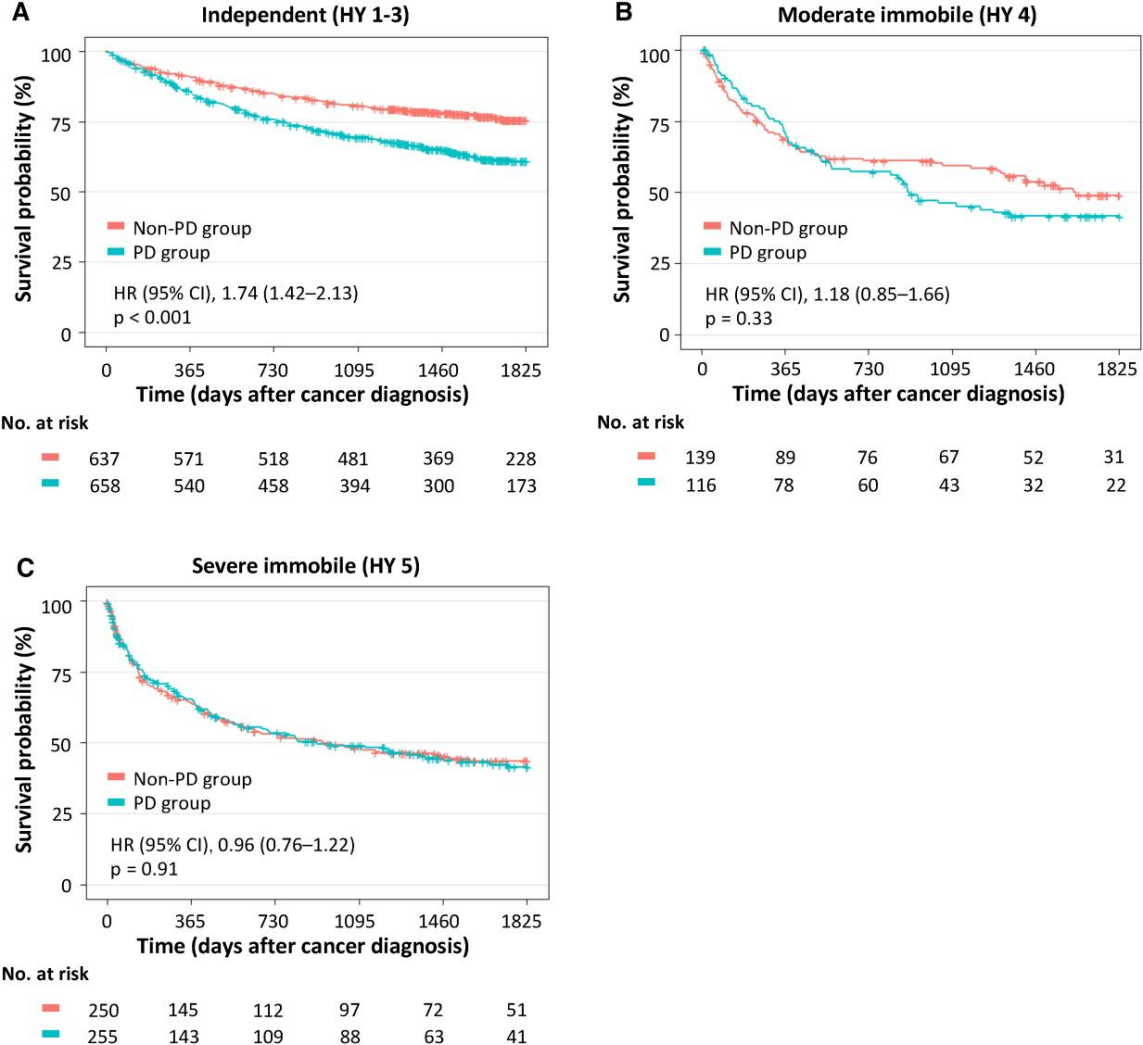
**Kaplan–Meier survival curves stratified by mobility status after propensity-score matching.** Kaplan–Meier survival curves of the Parkinson’s disease and non–Parkinson’s disease groups stratified by mobility status after propensity-score matching: independent mobility (**A**), moderate immobility (**B**) and severe immobility (**C**). The number of patients was 658 in the Parkinson’s disease group and 637 in the non–Parkinson’s disease group in group A and 116 in the Parkinson’s disease group and 139 in the non–Parkinson’s disease group in group B and 255 in the Parkinson’s disease group and 250 in the non–Parkinson’s disease group in group C. Among the patients with independent mobility, those in the Parkinson’s disease group showed poorer survival than those in the non–Parkinson’s disease group. For patients with moderate or severe immobility, survival rates were similar between the Parkinson’s disease and non–Parkinson’s disease groups.

**Table 3:** Baseline characteristics of cancer patients with and without Parkinson’s disease after propensity-score matching

Variables	PD group (*n* = 1049)	Non-PD group (*n* = 1049)	*P* value	SMD
Female, *n* (%)	430 (41)	443 (42)	0.56	0.025
Age, years (interquartile range)	73 (65–79)	72 (65–80)	0.61	0.02
<65	239 (23)	262 (25)		
≥65 and <75	345 (33)	362 (35)		
≥ 75	465 (44)	425 (41)		
Body mass index, *n* (%)			0.66	0.041
<18.5	165 (16)	168 (16)		
≥18.5 and <25	614 (59)	627 (60)		
>25	224 (21)	207 (20)		
Missing	46 (4)	47 (4)		
Smoking, *n* (%)	332 (32)	335 (32)	0.90	0.02
Charlson Comorbidity Index, *n* (%)			0.47	0.07
<3	422 (40)	417 (40)		
≥3 and <5	359 (34)	351 (33)		
≥5	266 (25)	281 (27)		
Missing	2 (0)	0 (0)		
Mobility, *n* (%)			0.98	0.019
Independent	658 (63)	658 (63)		
Moderate	116 (11)	113 (11)		
Severe	255 (24)	260 (25)		
Missing	20 (2)	18 (2)		
Cancer stage, *n* (%)			0.86	0.06
Intraepithelial	59 (6)	49 (5)		
Localized	452 (43)	454 (43)		
Regional	271 (26)	261 (25)		
Distant	182 (17)	194 (18)		
Not applicable	31 (3)	36 (3)		
Unstaged	54 (6)	55 (5)		
Cancer type, *n* (%)			0.96	0.002
Mouth-to-stomach	215 (20)	214 (20)		
Others	834 (80)	835 (80)		
Cancer treatment, *n* (%)				
Cancer surgery	640 (61)	621 (59)	0.40	0.037
Chemotherapy	289 (28)	283 (27)	0.77	0.013
Radiotherapy	107 (10)	101 (10)	0.66	0.06
Year of cancer diagnosis, *n* (%)			0.44	0.096
2010	41 (4)	29 (3)		
2011	85 (8)	102 (10)		
2012	167 (16)	150 (14)		
2013	213 (20)	214 (20)		
2014	286 (27)	285 (27)		
2015	257 (24)	269 (26)		

PD, Parkinson’s disease; SMD, standardized mean difference.

## Discussion

This retrospective multicentre cohort study investigated the influence of Parkinson’s disease on survival in >100 000 cancer survivors by using a population-based cancer registry linked to administrative data in Japan. The results showed that cancer patients with comorbid Parkinson’s disease had a significantly lower 5-year survival rate than those without comorbid Parkinson’s disease. Moreover, comorbid Parkinson’s disease was an independent predictor of mortality among cancer survivors. Although the Parkinson’s disease group consistently exhibited worse outcomes across nearly all measured parameters, including age and CCI score, compared to the non–Parkinson’s disease group, comorbid Parkinson’s disease remained an independent predictor of mortality even after adjusting for potential confounders. Several previous studies have revealed that comorbidity is an adverse prognostic factor for both Parkinson’s disease and cancer.^21,22^ However, few studies have investigated the effects of comorbid Parkinson’s disease on survival in cancer patients. To the best of our knowledge, no previous study has reported the effects of comorbid Parkinson’s disease on the long-term prognosis in cancer populations. The results of this study will provide important insights for clinicians managing patients with Parkinson’s disease and cancer.

We found that comorbid Parkinson’s disease is an independent predictor of mortality in patients with cancer. Several explanations can be provided for this finding. First, progression of Parkinson’s disease is linked to the duration of observation. Previous studies have indicated that Parkinson’s disease progresses naturally from HY3 to HY4 in over 90% of patients within approximately five years.^23,24^ Therefore, Parkinson’s disease progression during the observation period may have led to deterioration in motor function and death. Furthermore, Parkinson’s disease is known to worsen with anxiety.^25-27^ The diagnosis of cancer may have induced anxiety in patients, worsening their motor symptoms and potentially affecting their prognoses. Since the mouth-to-stomach cancer type was an independent poor prognostic factor in comparison with other cancers, the difficulty of managing Parkinson’s disease with oral medications during cancer treatment may have also influenced the survival rate. Additionally, patients in the Parkinson’s disease group may have experienced a poorer prognosis because of the lower rate of cancer treatment.

To further analyse the relationships of Parkinson’s disease and motor function with prognosis, we analysed the survival rates stratified by patient mobility. Patients with Parkinson’s disease who had independent mobility showed a poorer prognosis than non–Parkinson’s disease patients with independent mobility. In contrast, patients with moderate or severe mobility disabilities showed no difference in survival from non–Parkinson’s disease patients with moderate or severe mobility disabilities after cancer diagnosis. As mentioned earlier, in the group with independent mobility, Parkinson’s disease progression may have been associated with death during the study period. Notably, the mobility measurements in our study were obtained from administrative data and were not assessed over time. In addition, we estimated motor symptoms using the Barthel index because there was no Parkinson’s disease -specific index such as the HY scale in the database. We believe that direct and longitudinal neurological assessment can improve the accuracy of the analysis of whether Parkinson’s disease has progressed. A cause-of-death analysis would also be useful in considering the impact of Parkinson’s disease on cancer patients. Since the cause of death was not included in this database, we plan to conduct such a study in the future. Findings from these future analyses may also contribute to the elucidation of biological pathways. Recently, several biological mechanisms have been reported to be common to both cancer and Parkinson’s disease. For example, dysfunction in mitochondrial quality control may serve as a common aetiological factor for both cancer and Parkinson’s disease. Pathological mutations in *PARK2*, a causative gene for autosomal recessive juvenile Parkinson’s disease, result in loss of function of parkin, a protein involved in mitochondrial quality control such as mitophagy. These mutations have been also implicated in carcinogenesis.^28^ However, other studies have found no association between *PARK2* mutations and cancer risk,^29^ making the role of parkin in tumorigenesis controversial and in need of further investigation. Exercise is also recognized as a key intervention for maintaining and improving symptoms in patients with Parkinson’s disease, and it has also been shown to attenuate disease progression. For example, one proposed mechanism involves irisin, a myokine released during physical activity, which has been reported to suppress alpha-synuclein pathology and exert neuroprotective effects in Parkinson’s disease animal models.^30^ Both Cancer and Parkinson’s disease may lead to decreased activity and performance status, which may result in a loss of beneficial benefits from exercise and may affect the course of the disease. Further elucidation of underlying mechanisms through both clinical and basic research is warranted.

The current study also demonstrated that a more recent cancer diagnosis was associated with a decreased risk of mortality. This can be attributed to advances in cancer care and Parkinson’s disease therapeutic interventions. For instance, ipilimumab was approved as the world’s first immune checkpoint inhibitor for the treatment of malignant melanoma in 2011. In Japan, following the approval of nivolumab for malignant melanoma in 2014, several immune checkpoint inhibitors have been increasingly utilized, leading to advancements in cancer treatment.^31^ In terms of Parkinson’s disease therapy, the number of available Parkinson’s disease treatment drugs has substantially increased in Japan since the registration of this study cohort began in 2010. For example, pramipexole extended-release (approved in 2011), ropinirole extended-release (approved in 2012), rotigotine patch (approved in 2013) and ropinirole patch (approved in 2019) enable continuous dopamine stimulation by dopamine agonists. Rasagiline (approved in 2018) and safinamide (approved in 2019) have expanded the choices of monoamine oxidase B (MAO-B) inhibitors. Istradefylline (approved in 2013) improves symptoms by antagonizing adenosine signalling. Ultra-short-acting apomorphine injections (approved in 2012) and levodopa-carbidopa intestinal gels (approved in 2016) have also been approved for clinical use. Therefore, more recently diagnosed individuals may have benefited from the broader range of treatment options available for both diseases.

Our study also emphasizes the importance of collaboration between oncologists and neurologists. Oncologists should be aware of the comorbidities in their patients. In patients with cancer and comorbid Parkinson’s disease, especially when mobility is maintained, consultation with a neurologist is recommended to maintain good control of Parkinson’s disease symptoms, since this can affect the patients’ prognosis and therapeutic decision-making based on their performance status. When adequate control of parkinsonian symptoms is difficult with oral medication, particularly for some patients with mouth-to-stomach cancers, transdermal patches and device-assisted therapies are good choices for stabilizing parkinsonian symptoms. Notably, our study did not assess neurologist interventions for the treatment of Parkinson’s disease. Since neurologist care of patients with Parkinson’s disease may be associated with improved clinical outcomes and survival,^32^ further research is warranted to determine whether the prognosis of patients with cancer and Parkinson’s disease improves through treatment interventions by neurologists.

The strength of this study is its extensive investigation of Parkinson’s disease and cancer over a 5-year period by utilizing OCR data in Japan. However, this study had several limitations. First, the assignment of Parkinson’s disease was based on the ICD-10 code from the DPC data, leading to the possibility of misclassification. To address this issue, patients were defined as having Parkinson’s disease based on the disease code as well as the prescription of Parkinson’s disease drugs, including L-dopa, dopamine agonists and MAO-B inhibitors. Therefore, we believe that the influence of misclassifications was limited. Second, this study did not consider the duration of Parkinson’s disease, which may be associated with the outcomes. Third, we did not have information on income or detailed cancer treatment regimens that could affect survival outcomes. Although Japanese universal health insurance system allows people to receive unlimited medical care regardless of their economic status, socioeconomic status might affect prognosis because it influences the living environment and behavioural patterns.

This study reported that patients with cancer and comorbid Parkinson’s disease had a worse prognosis than those without comorbid Parkinson’s disease. Survival analysis stratified by mobility revealed that the prognosis was poorer in patients with independent mobility. Thus, Parkinson’s disease care after cancer diagnosis is important, and Parkinson’s disease treatment should receive adequate attention along with cancer treatment in patients with cancer and comorbid Parkinson’s disease.

## Supplementary Material

fcaf347_Supplementary_Data

## Data Availability

Anonymized data not published within this article will be made available by request from any qualified investigator. We showed the R code in Supplementary Data 1.
