# Supplementary material for: Effects of Parkinson’s disease on survival in cancer survivors: a retrospective, multicentre cohort study in Japan
Source: Brain Commun. 2025 Sep 13;7(5):fcaf347. doi: 10.1093/braincomms/fcaf347 (PMC12492484; doi:10.1093/braincomms/fcaf347)
Supplement: fcaf347_Supplementary_Data [file fcaf347_supplementary_data.docx]

**SUPPLEMENTARY MATERIAL**

**Effects of Parkinson disease on survival in cancer survivors: a retrospective, multicenter cohort study in Japan**

**Data S1. R code**

**Table S1. The assignment of codes and the number of patients**

**Table S2. Disease diagnosis codes and antiparkinsonian medication classification**

**Data S1. R code**

### Install libraries

library(rms)

library(ggsurvfit)

library(survival)

library(Matching)

**Table 1. Baseline characteristics of patients with cancer with and without Parkinson disease**

summary(pd ~ variable, method="reverse", overall=F, test=T, data=dataset)

# pd=1 (patients with Parkinson disease)

# variable=female, age, …, years of cancer diagnosis

**Table 2. Hazard ratios for mortality determined using Cox proportional-hazard models**

### Univariable

coxph(Surv(days, death==1) ~ variable, data=dataset, method="breslow")

# variable=female, age, …, years of cancer diagnosis

### Multivariable

dd <- datadist(dataset)

options(datadist='dd')

set.seed(seed=1)

form=~var1+var2+var3+…varx

areg=aregImpute(formula=form, data=dataset, n.impute=5, nk=3, tolerance=1e-99)

fit=fit.mult.impute(Surv(days, death==1) ~ variables, coxph, xtrans=areg, data=dataset)

summary(fit)

# variable=pd+male+age+…+years of cancer diagnosis

**Table 3. Baseline characteristics of cancer patients with and without Parkinson disease after propensity-score matching**

### Propensity score matching

set.seed(seed=1)

areg = aregImpute(pd ~ variables, data=dataset, n.impute=5, x=TRUE)

fit.mi <- fit.mult.impute(pd ~ variables, lrm, areg, data=dataset)

dataset$linear.ps <- fit.mi$linear.predictors

dataset$p = exp(dataset$linear.ps/(1+exp(dataset$linear.ps)))

match.results=Match(Y=NULL, Tr=(dataset$pd==1), X=dataset$p, M=1, caliper=0.25, ties=FALSE, replace = FALSE)

pairmatch.treated <- 1:length(match.results1$index.treated)

pairmatch.control <- rep(pairmatch.treated, each=1)

pairmatch <- c(pairmatch.treated, pairmatch.control)

match.data <- rbind(dataset[match.results$index.treated,],dataset[match.results$index.control,])

### Comparison between two groups

summary(pd ~ variable, method="reverse", overall=F, test=T, data = match.data)

# pd=1 (patients with Parkinson disease)

# variable=female, age, …, years of cancer diagnosis

**Figure 1. Kaplan-Meier survival curves stratified by the presence of Parkinson disease**

fit <- survfit(Surv(days, death) ~ pd, data=dataset)

p.all <- ggsurvplot(fit, data=dataset, risk.table=T, risk.table.type ="absolute", tables.theme=theme_cleantable(), risk.table.y.text=FALSE, risk.table.col="black", risk.table.height=0.25, xlim=c(0,1826.25), break.time.by=365, ggtheme=theme_base())

p.all

**Figure 2. Kaplan-Meier survival curves stratified by mobility status**

# Independent (HY 1-3)

fit <- survfit(Surv(days, death) ~ pd, data = subset(dataset, adl==”independent”)

p.ind <- ggsurvplot(fit, data= subset(dataset, adl==”independent”, risk.table=T, risk.table.type="absolute", tables.theme=theme_cleantable(), risk.table.y.text=FALSE, legend="none", risk.table.col="black", risk.table.height=0.25, conf.int=FALSE, xlim=c(0,1826.25), break.time.by=365, ggtheme=theme_base())

p.ind

# Moderate immobile (HY 4)

fit <- survfit(Surv(days, death) ~ pd, data = subset(dataset, adl==”immobile”)

p.imm <- ggsurvplot(fit, data=subset(dataset, adl==”immobile”), risk.table=T, risk.table.type="absolute", tables.theme=theme_cleantable(), risk.table.y.text=FALSE, legend="none", risk.table.col="black", risk.table.height=0.25, conf.int=FALSE, xlim=c(0,1826.25), break.time.by=365, ggtheme=theme_base())

p.imm

# Dependent (HY 5)

fit <- survfit(Surv(days, death) ~ pd, data=subset(dataset, adl==”dependent”)

p.dep <- ggsurvplot(fit, data= subset(dataset, adl==”dependent”), risk.table=T, risk.table.type="absolute", tables.theme=theme_cleantable(), risk.table.y.text=FALSE, legend="none", risk.table.col="black", risk.table.height=0.25, conf.int=FALSE, xlim=c(0,1826.25), break.time.by=365, ggtheme=theme_base())

p.dep

**Figure 3. Kaplan-Meier survival curves stratified by the presence of Parkinson disease after propensity score matching**

fit <- survfit(Surv(days, death) ~ pd, data=match.data)

p.mtc <- ggsurvplot(fit, data=match.data, risk.table=T, risk.table.type="absolute", tables.theme=theme_cleantable(), risk.table.y.text=FALSE, legend="none", risk.table.col="black", risk.table.height=0.25, conf.int=FALSE, xlim=c(0,1826.25), break.time.by=365, ggtheme=theme_base())

p.mtc

**Figure 4. Kaplan–Meier survival curves stratified by mobility status after propensity-score matching**

# Independent (HY 1-3)

fit <- survfit(Surv(days, death) ~ pd, data=subset(match.data, adl==”independent”)

p.ind <- ggsurvplot(fit, data= subset(match.data, adl==”independent”), risk.table=T, risk.table.type="absolute", tables.theme=theme_cleantable(), risk.table.y.text=FALSE, legend="none", risk.table.col="black", risk.table.height=0.25, conf.int=FALSE, xlim=c(0,1826.25), break.time.by=365, ggtheme=theme_base())

p.ind

# Moderate immobile (HY 4)

fit <- survfit(Surv(days, death) ~ pd, data=subset(match.data, adl==”immobile”)

p.imm <- ggsurvplot(fit, data=subset(match.data, adl==”immobile”), risk.table=T, risk.table.type="absolute", tables.theme=theme_cleantable(), risk.table.y.text=FALSE, legend="none", risk.table.col="black", risk.table.height=0.25, conf.int=FALSE, xlim=c(0,1826.25), break.time.by=365, ggtheme=theme_base())

p.imm

# Dependent (HY 5)

fit <- survfit(Surv(days, death) ~ pd, data=subset(match.data, adl==”dependent”)

p.dep <- ggsurvplot(fit, data=subset(match.data, adl==”dependent”), risk.table=T, risk.table.type="absolute", tables.theme=theme_cleantable(), risk.table.y.text=FALSE, legend="none", risk.table.col="black", risk.table.height=0.25, conf.int=FALSE, xlim=c(0,1826.25), break.time.by=365, ggtheme=theme_base())

p.dep

**Table S1. The assignment of codes and the number of patients**

|  | ICD-O-3 morphology | ICD-O-3 | N | % |
| --- | --- | --- | --- | --- |
| All |  |  | 118,999 | 100% |
| Lip, oral cavity, and pharynx |  | C00-C14 | 1,368 | 1.1% |
| Esophagus |  | C15 | 3,553 | 3.0% |
| Stomach |  | C16 | 15,267 | 12.8% |
| Colorectal |  | C18-C20 | 19,176 | 16.1% |
| Liver and intrahepatic bile duct |  | C22 | 6,187 | 5.2% |
| Gallbladder and other biliary tract |  | C23-24 | 2,454 | 2.1% |
| Pancreas |  | C25 | 4,710 | 4.0% |
| Larynx |  | C32 | 866 | 0.7% |
| Trachea, bronchus, and lung |  | C33-C34 | 13,235 | 11.1% |
| Bone and soft tissue |  | C40, C41, C47, C49 | 1,074 | 0.9% |
| Skin |  | C44 | 2,507 | 2.1% |
| Breast |  | C50 | 9,794 | 8.2% |
| Uterus |  | C53, C54 | 6,445 | 5.4% |
| Ovary |  | C56 | 1,368 | 1.1% |
| Prostate |  | C61 | 9,672 | 8.1% |
| Bladder |  | C67 | 4,647 | 3.9% |
| Renal and urinary tract |  | C64-C66, C68 | 3,325 | 2.8% |
| Brain |  | C700, C71, C751-753 | 1,705 | 1.4% |
| Thyroid |  | C73 | 1,418 | 1.2% |
| Hematologic | 959-976, 980-998 |  | 6,465 | 5.4% |
| Other malignant neoplasms |  | Others | 3,268 | 2.7% |

ICD-O-3, International. Classification of Diseases for Oncology, 3rd Edition.

**Table S2. Disease diagnosis codes and antiparkinsonian medication classification**

|  | ICD–10 |
| --- | --- |
| Parkinson disease | G20 |
| Cerebrovascular disease | I60-69 |
| Myocardial infarction | I21 |
| Peripheral arterial occlusion | I74 |
| Dementia | F00-03 |
| Diabetes mellitus | E10–E14 |
| Heart failure | I50, I110, I130, I132 |
| Chronic obstructive pulmonary disease | J40–47, J60–J67, J684, J701, J703, J841, J920, J961, J982, J983 |
| Renal disease | E102, E112, E142, N03, N05, N110, N14, N16, N18, N19, N269 Q611–Q614 |
| Liver disease | K70–K77 |
| Acquired immunodeficiency syndrome | B20-B24 |
| Hemiparalysis | G81 |
|  | Drug names |
| Antiparkinsonian drugs | |
| Levodopa | Levodopa |
| Levodopa/dopa decarboxylase inhibitors | Levodopa/benserazide, levodopa/carbidopa, levodopa/carbidopa/entacapone |
| Dopamine agonists | Bromocriptine, cabergoline, pergolide, pramipexole, ropinirole, rotigotine, apomorphine |
| Monoamine oxidase B inhibitors | Selegiline, rasagiline |

ICD-10, International. Classification of Diseases, 10th Edition
